# Rodent models of postherpetic neuralgia: How far have we reached?

**DOI:** 10.3389/fimmu.2023.1026269

**Published:** 2023-03-20

**Authors:** Mingxi Ou, Jiamin Chen, Shaomin Yang, Lizu Xiao, Donglin Xiong, Songbin Wu

**Affiliations:** ^1^ Department of Pain Medicine and Shenzhen Municipal Key Laboratory for Pain Medicine, Huazhong University of Science and Technology Union Shenzhen Hospital, Shenzhen, China; ^2^ Department of Chemistry, University of Science and Technology of China, Hefei, China; ^3^ Teaching and Research Group of Biology, Vanke Bilingual School (VBS), Shenzhen, China

**Keywords:** postherpetic neuralgia, varicella zoster virus, Herpes simplex virus 1, pain assessment, pathogenesis

## Abstract

**Background:**

Induced by varicella zoster virus (VZV), postherpetic neuralgia (PHN) is one of the common complications of herpes zoster (HZ) with refractory pain. Animal models play pivotal roles in disclosing the pain mechanisms and developing effective treatments. However, only a few rodent models focus on the VZV-associated pain and PHN.

**Objective:**

To summarize the establishment and characteristics of popular PHN rodent models, thus offer bases for the selection and improvement of PHN models.

**Design:**

In this review, we retrospect two promising PHN rodent models, VZV-induced PHN model and HSV1-induced PHN model in terms of pain-related evaluations, their contributions to PHN pathogenesis and pharmacology.

**Results:**

Significant difference of two PHN models is the probability of virus proliferation; 2) Most commonly used pain evaluation of PHN model is mechanical allodynia, but pain-induced anxiety and other behaviours are worth noting; 3) From current PHN models, pain mechanisms involve changes in virus gene and host gene expression, neuroimmune–glia interactions and ion channels; 4) antiviral drugs and classical analgesics serve more on the acute stage of herpetic pain.

**Conclusions:**

Different PHN models assessed by various pain evaluations combine to fulfil more comprehensive understanding of PHN.

## Introduction

Varicella-zoster virus (VZV) is one kind of human neurotropic viruses (1). Primary infection with VZV usually causes chickenpox and establishes the latent infection in the dorsal root ganglia. In the elderly or people with weakened immune systems, reactivation of dormant VZV may occur and lead to herpes zoster (HZ), which is one type of acute local herpes-like skin lesions. 5% to 30% of HZ patients may suffer from severe pain persisting for more than 3 months after recovery from the skin lesions, referred to as post-herpetic neuralgia (PHN) (2).

To date, two vaccines for VZV infection are available: a live-attenuated vaccine and a new adjuvant recombinant VZV vaccine (3). However, their prevention of PHN has not achieved an ideal effect and lacks of long-term post-marketing surveillance. After zoster onset, no effective treatment is available for preventing or delaying PHN. Although antiviral medications combined with steroids such as prednisone have shown the potential to lower the risk of PHN, the period of beneficial administration is short after rash onset (4). It requires a rapid diagnosis in the early state of HZ or virus reactivation, but it is difficult to be reached in clinics. Even the HZ patients get the rapid diagnosis and treatment, the effect is often modest on the prevention and alleviation of the associated pain (5). The debilitating and refractory pain leads to physical disability, poor sleep, and psychosocial dysfunction of PHN sufferers, which interferes with their normal daily activities and raises a burden on their families (6, 7). On account of the increasing aging population worldwide, the incidence of HZ and PHN presents a visibly growing trend over the next decades (8). The prevention and treatment of PHN have been an urgent issue.

Appropriate animal models are required to explore PHN pathogenesis and support the examination of new prevention and treatment strategies. Although several animal models of VZV infection have been published and recapitulated to help understand VZV biology and HZ onset, there is only a few rodent models focusing on the VZV-associated pain and PHN. In 1999, behavioural changes suggestive of abnormal pain state in VZV-infected rat models was firstly described (9). Over the decades, scientists pay increasing attention on the development of VZV-associated pain and PHN, and some rodent models have been developed to figure out mechanism behind the persistent pain and verify novel treatments and managements (10–12). However, these models fail to mimic all clinical features seen in PHN patients. One of the notable features is the failure to induce rash onset in VZV-injected mouse models. Moreover, due to the strict human specificity, VZV persistence in animal models is elusive (13), hindering the study of the relationship between virus proliferation and herpes-related pain. To solve this difficulty, alternative modelling methods have been attempted to study VZV infection, including 1) VZV-infection of severe-combined immunodeficient (SCID) mouse model implanted with human fetal tissues (14); 2) inoculation of rhesus macaques with Simian Varicella Virus (SVV, a homologous virus causing vesicular rash in Old World monkeys) (15); 3) inoculation of herpes simplex virus 1 (HSV1, a similar neurotropic virus with VZV) (16, 17). Among the above modelling methods, HSV-1 infection was more commonly used because of the ability to mimic natural virus transmission and the ease of maintenance. HSV-1 infection has been reported to induce PHN-like herpetic pain in rodent models (16, 17). In this review, we mainly discuss the two most popular rodent models of VZV or HSV1-induced herpetic pain, their potential for understanding possible pathogenesis and pharmacology as well as their limitations.

## Virological features of VZV and HSV1

Varicella-zoster virus (VZV) and herpes simplex virus 1 (HSV1) are human neurotropic alpha-herpesviruses that often acquired early in life (18). Following primary infection, VZV and HSV1 both establish a latency in human peripheral ganglia. VZV is the causative agent of varicella (chickenpox) and its reactivation results in herpes zoster (HZ, or shingles), while latent HSV1 may reactivate repeatedly to produce herpes labialis typically.

VZV particles are pleomorphic to spherical in shape, about 150 ~ 200 nm in diameter and are composed of three protein layers: a nucleocapsid containing the viral double stranded DNA (dsDNA) genome, a tegument layer surrounding the nucleocapsid, and an envelope with viral glycoproteins facing outwards (1). The core has been reported as a loose fibrillar cage of strands that surround a dense cylindrical core of DNA fibers (19). VZV genome is the smallest of the human herpesviruses at about 125 kilo base (kb) pairs and consists of a unique long region (UL) flanked by terminal long (TRL) and internal long (IRL) repeats, and a unique short region (US) bounded by internal short (IRS) and terminal short (TRS) repeats (20). Since the US region can orientate either of two directions, while the UL region rarely changes its orientation, there are usually two isomers of the genome in infected cells. Although the UL region of HSV1 is orientated in the opposite direction to that of VZV, they are largely collinear. The VZV US region is much shorter (5.2 kb) than HSV-1 US (13.0 kb), and the TRL and IRL regions are also shorter (0.9 kb) than the HSV-1 counterparts (9.2 kb).

In most tissue culture cells, HSV1 is an efficient virus that can overcome the species specificity, whose lytic replication cycle culminates in 8 ~ 12 h. Although the VZV lytic replication cycle culminates in 9 ~ 12 h (21), it can take 3 ~ 5 days for the entire culture to show extensive VZV-induced syncytia and cell death either by lysis or apoptosis (22). Due to the highly cell-associated property of VZV (23), it is hard to quantify the viral titer in the harvested VZV-infected cell, adding difficulties to reproduce the results in VZV-induced PHN models (**Table 1**).

**Table 1:** PHN models and pain evaluation.

Models		VZV-induced herpes related pain	HSV-1-induced herpes related pain
**Model establishment**	**Rodent animals**	Sprague-Dawley rats (11, 12, 24–26); Wistar rats (27–31); Long Evans rats (12); C57BL/6J mice (32)	C57BL/6J mice (33–44); BALB/c (35, 45–47)mice;
	**Virus infection**	Cells harvested 48~72 hours post infection of VZV	HSV-1 suspension
	**Virus inoculum**	25-50 μL TRPE cell suspension containing 2×10 (5) PFU VZV strain pOka (24, 26, 27); 100 μL MeWo cell suspension containing 3.6×10 (5)-1.9×10 (6) PFU) VZV strain pOka (12) (11); 65 μL MRC-5 cells containing 3.2×10 (7) or 6.4×10 (5) PFU/μL VZV strain KMcC (29); 50 μL CV-1 cell suspension (4-8×10 (6) cells infected with VZV strain Ellen) (25, 28, 30, 31)	1×10 (6) PFU HSV-1 (7401H strain) (35, 45, 48, 49); 2×10 (5) PFU HSV-1 (34, 36).
	**Inoculation site**	Rear footpad (24–28, 30, 31); Whisker pad (11, 12, 27)	Right skin epidermis (35, 50); Skin of right hind paw (33, 34, 36, 38–44, 47, 51–56);
**Characteristics**	**Viral genome or protein**	IE62 (12, 24, 29); ORF62/63/47/29 (24)	gB (35); HSV-1 polymerase gene (34); TK (51)
	**Herpetic lesions**	None	Typical appearance of herpes zoster-like skin lesions (33–37, 39, 41, 44, 51)
**Pain evaluation**	**Behavioral tests**	Mechanical hyperalgesia (12, 24–31); Thermal hyperalgesia (12, 24, 26, 27, 29); Aversive behavior (11, 12);	Mechanical hyperalgesia (12, 24–31); Thermal hyperalgesia (12, 24, 26, 27, 29); Aversive behavior (11, 12);
	**Initiation time of pain-related behaviors**	Day 3 - 21 after vaccination (24) (27, 30–32) Mostly from day 7 after vaccination (11, 12) (25–29, 31)	Day 3 (33–35); Day 5 (11, 25, 35, 45, 47, 57, 58); Day 6 (40, 56); Day 7 after vaccination;
	**Maximum time to monitor pain-related behaviors**	28 days (25); 35 days (26); 43 days (24); 49 days (12); 63 days (32); 66 days (27); 70 days (28, 29); 90 days (27) after vaccination	20 days (39); 21 days (34, 36); 30 days (35) 37 days (38); 42 days (33); 50 days (40) after vaccination

VZV, varicella zoster virus; HSV1, herpes simplex virus 1; PFU, plaque-forming unit.

Upon VZV virion entering the host cell, tegument proteins are released into the newly infected cell and alter the host environment, thereby inhibiting antiviral responses and influencing the virus program, that is, a lytic or latent infection. During productive infection the complete VZV proteome consisting of some 68 unique gene products is expressed through interaction of a small number of viral transcriptional activators with the general transcription apparatus of the host cell. VZV virion proteins delivered into newly infected cells upon entry are not absolutely required to initiate VZV gene expression, as evidenced by the resulting VZV replication upon transfection of cells with viral DNA. It has been reported that protein-free HSV-1 DNA is also infectious when properly introduced into cells, albeit at low efficiencies. However, the relationship between virion gene expression and VZV-associated pain remains unclear.

## The development of PHN rodent models and pain evaluations

### VZV-infected models paved the way

**Figure 1:**
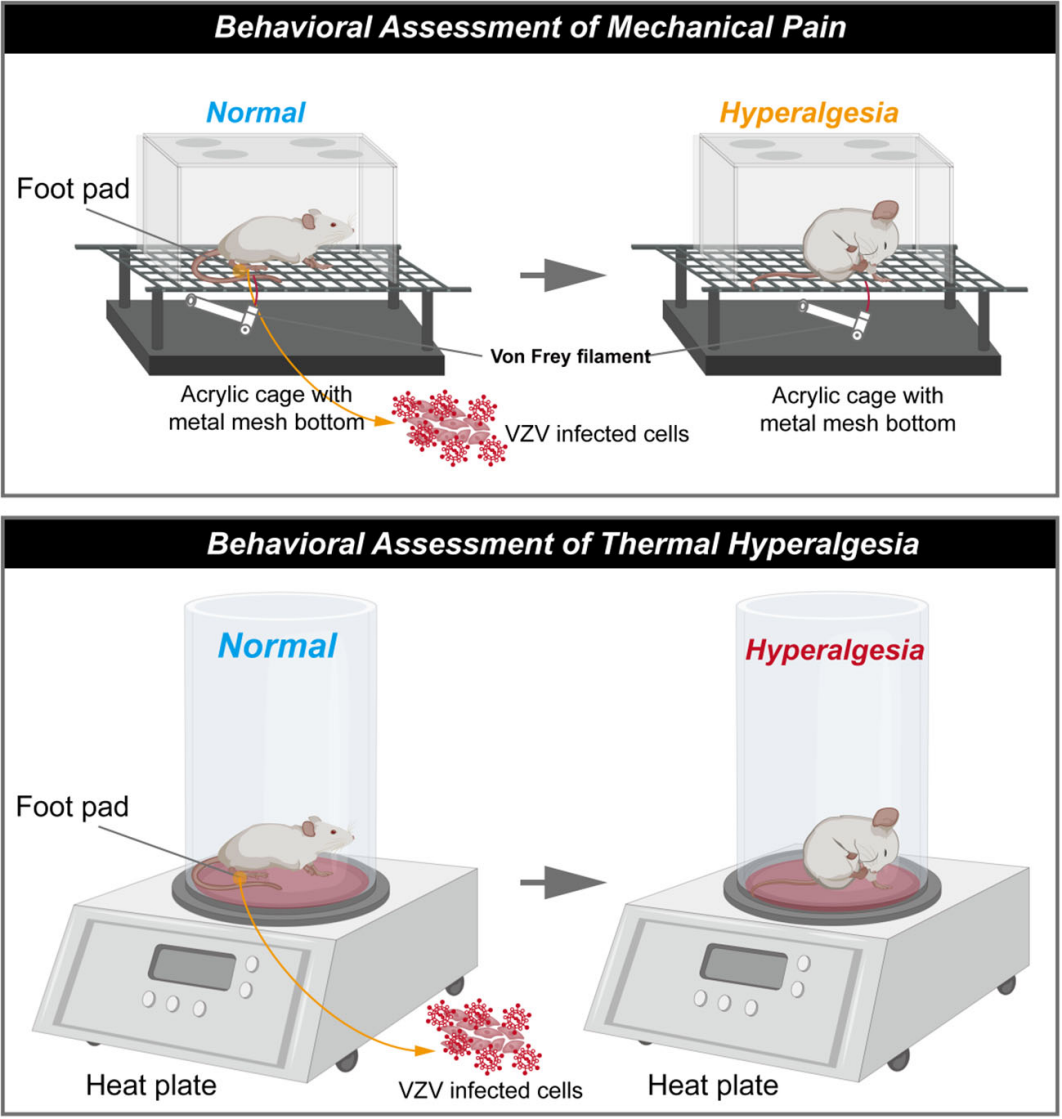
Schematic diagram of rat model of postherpetic neuralgia induced by VZV. (The schematic was drawn by Figdraw, https://www.figdraw.com/static/index.html#/).

Fleetwood-Walker’s group first described prolonged pain-related behaviour from VZV-infected rats (shown in **Figure 1**). The infected rats exhibited significant changes in behavioural responses indicating mechanical allodynia and hyperalgesia (9) for up to 33 days post-injection. These symptoms continue after all evidence of local injection trauma had disappeared (meaning that the abnormal behaviours were not caused by inflammatory response). However, Fleetwood-Walker’s group stopped the behavioural tests while the sensory abnormalities did not return to the normal values on the 33^rd^ day following injection (9).

This PHN model has been extended by others, indicative that the pain state lasts for a longer time, and in behaviour tests measuring affective pain responses (12, 24, 27–29, 32). Rats consistently developed mechanical hyperalgesia from the first week following injection (12, 25–32), and the pain lasted for several weeks, and gradually relieved within 90 days after VZV injection. Mechanical allodynia has been proved to be irrelevant by viral strains but in a dose-dependent pattern during pain development (10). However, no shingles or skin lesions have been observed in VZV-induced PHN models (45), which is one of important clinical features observed in humans. The outbreak of shingles associates with cell-mediated immunity after VZV reactivation and replication, and it seems to explain why VZV-infected models fail to induce analogous lesions: although VZV DNA and the transcripts have been detected in several cells and organs (48), the virus is unable to replicate in the rodent due to the species specificity.

In sum, rodent models of VZV infection performed pain-associated behaviour, paving the way for developing the PHN models. The modelling is usually required to harvest VZV propagated on human cell lines and subcutaneously injected in rats’ unilateral glabrous footpad (12, 24, 27–29, 32), characterized by mechanical hyperalgesia. To further optimize PHN modelling, two problems are noteworthy: (a) VZV fail to replicate in rodent animals as humans are its specific natural host; (b) pain is hard to measure in non-communicating animals.

### HSV-1 mice models provide a solution to the species specificity

**Figure 2:**
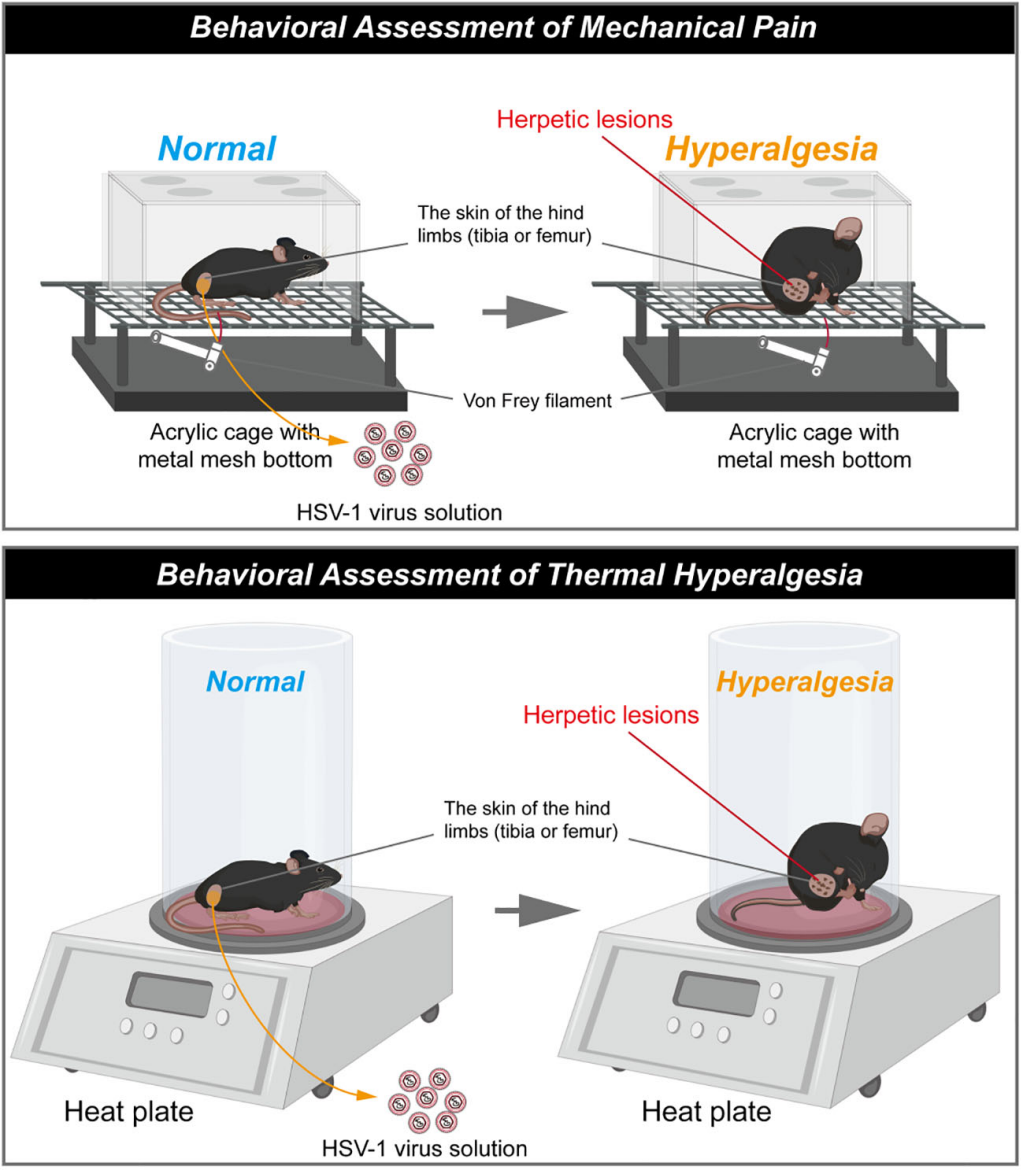
Schematic diagram of herpes associated pain model induced by HSV1. (The schematic was drawn by Figdraw, https://www.figdraw.com/static/index.html#/).

VZV inoculation enables rats to develop mechanical allodynia and thermal hyperalgesia, but no shingles or skin lesions are observed (45). As we discussed in last section, HSV1 is highly infectious to mice and can replicate in various organs, which overcome species specificity (46). As with VZV, HSV1 establishes latency in the sensory ganglia, and the reactivation causes herpes simplex over again after the primary infection. In this process, pain occurs before the outbreak of herpes, or even after the vesicles disappear (16, 17). What makes HSV1-induced PHN model different is that transdermal HSV1 inoculation induces shingles-like skin lesions in the inoculated skin and back of the mice (shown in **Figure 2**). Thus, HSV-1 infected mice models provide a solution to the loss of skin lesion after herpetic virus inoculation and the chance to study the relationship between herpetic virus proliferation and herpes-related pain.

The skin lesions generally last for 20 days and then dissipate (33, 34, 51). Meanwhile, HSV1 inoculation also induce mechanical allodynia and hyperalgesia in the hind paws of mice as with VZV (33, 59). It has been reported that obvious herpetic neuralgia generally occurred on day 5 post inoculation of HSV1 and peaked on the day 7 post inoculation, then the pain would be gradually relieved. However, the mechanical allodynia and mechanical hyperalgesia generally last for 21 days (34, 36). In this model, the thermal hyperalgesia are not observed during the outbreak and presence of herpes, but was induced in the post-herpes phase (49).

### Methods used to evaluate pain in rodent animals

As pain is hard to be measured directly in rodent animals, some typical methods have been developed to observe and quantify pain-induced behaviours or nociception in PHN rodent models (**Table 2**). Generally, evoked pain response after virus (VZV or HSV1) infection are measured. The most popular tests to characterize PHN model include mechanical stimuli using manual or electronic Von Frey and temperature stimuli using heat or cold plate test. The limitation concerning these tests is that the measurement usually restricts to the paw of rodents, ignoring the back pain or orofacial that may occur in PHN patients. Given that about a quarter of PHN patients suffer from orofacial pain (12), orofacial pain model has been established by VZV injection into rats’ whisker pad and assessed by escape and/or avoidance behaviour (Place Escape/Avoidance Paradigm) (11, 12).

**Table 2:** Methods that applied in the characterization of current PHN rodent models.

Changed behaviour	Behavioural test	Equipment	Measurement
Mechanical allodynia	von Frey test; dynamic mechanical stimulation	Cages with a metal mesh grid floor (32) von Frey filament (11, 24, 26–31); electronic von Frey filament (25); paintbrush (33, 35); cotton bud	The reaction time of paw withdrawal from the filament, jumping, or licking the toes
Thermal allodynia	Heat or cold plate test (32)	Hargrave’s apparatus (the core is a metal plate providing heat or cold stimulation) (24, 26, 27, 29)	The reaction time of paw withdrawal from the hot plate, licking, shaking the hind paw, jumping, or spinning
Reduced time spent on drinking due to orofacial pain	Orofacial test chamber from Ugo Basile (12)	reward such as a sweetened condensed milk mixture	Time spent drinking
Reduced percentage of time on the dark side	Fuchs place-escape-avoidance-paradigm (PEAP) (11, 12, 27)	An acrylic box half covered in black cloth	Percentage of time on the dark side, reduced time indicates a greater affective pain response
more thigmotactic ambulation (wall-hugging) less frequently entered the central area of the open field arena	Open field test (10)	a square plastic arena camera	Frequency of entry into and time spent in the center of open field arena, total distance travelled

Nevertheless, the behavioural tests may simplify the pain state in rodent animals. And evoked pain related experiments introduce man-made interference and operations that may trigger stress-induced analgesia in rodent animals, confusing the results of pain evaluation. It is notable that PHN patients also suffer from spontaneous pain that do not require an identifiable stimulus (60) and persisting pain may induce depression and anxiety. In the open field paradigm, anxiety-like patterns of ambulation has been observed in the virus-infected rats, which is positively correlated with mechanical hypersensitivity (shown in **Table 2**) (10). This behavioural feature assists us in understanding some mental states of PHN sufferers, and provide an additional aspect to characterize the rodent models of PHN. Besides, spontaneous pain can be evaluated by grimace scales, eye closure or eye blinking, gait analysis, food, or water consumption, etc (60), which methods have been applied in other pain models like tooth injury (61), but PHN rodent animals lack related information. More pain-induced performance should be combined with the normally used tests to comprehensively assess the pain state of rodent animals and their relationship with some molecular changes of pain indicators need to be further explored. These supplementary data will complement the characterization of PHN models and help mimic the pain-associated comorbidity of PHN patients.

## PHN pathogenesis learned from rodent models

The latency and activation of VZV are incentive of PHN, but the transition from VZV activation to PHN remains elusive. Through current rodent models, some mechanisms of PHN onset have been gradually emerging from behind the fog. Here, we summarized the roles of some factors in the PHN pathogenesis.

### Virus infection

Different from the other neuropathic pain, VZV establishes a latent infection in the host. No evidence proved that VZV could proliferate in the nervous system (49), but viral DNA and transcripts have been detected in the DRG of the rodents after infection (34, 51). Some reports suspect that transcription of VZV genes within virus-infected neurons may lead to the chronic pain, either through induction of inflammatory mechanisms or through expression of proteins, such as transcriptional regulators that alter host cell expression patterns. VZV gene 63 is suggested to be important for the maintenance of virus latency because of the high abundance of its gene transcripts (62). As for gene regulators, IE62 and IE63 proteins are detected in the VZV-infected rats’ DRG, and IE62 co-expressed with the marker of A- and C- afferent sensory neurons. This events also link with an increased expression of neuropeptide Y (NPY), which has been implicated in both pro- and antinociceptive effects (63). In the HSV-1 mice models, viruses are able to proliferate in the DRG and primary sensory neurons and viral genome can be detected in 20% to 30% of DRG neurons (50, 64).The amount of HSV-1 DNA significantly increases from day 4 to 7 post-inoculation, and then followed with a rapid decline (51) (34). During this period, shingles begin to erupt in the skin of the inoculated dermatome.

### Inflammatory responses

Initially, the immune response has been considered as a key factor in zoster-associated pain, as shingles are linked with inflammation and haemorrhagic necrosis of the ganglia with accompanying degeneration of motor and sensory roots (65, 66). However, PHN is somewhat resistant to opioid and non-steroidal anti-inflammatory drugs, the mainstay of treatments for acute and inflammatory pain states, implying that inflammatory responses are not sufficient to induce PHN.

### Changes in nervous systems

Clinical evidence indicates that PHN may be caused by altered excitability of ganglionic or even spinal cord neurons, particularly neuronal hyperexcitability in damaged areas of the nervous system (67).The footpads of VZV-induced PHN rats showed changes in neuronal populations and reduced peripheral innervation and changes in neuronal populations. In the scarred skin and DRG of HSV1-induced pain models, C-fiber neurons are significantly reduced, but A-fiber neurons remains unchanged (29, 64). These results suggest that post-herpetic dynamic allodynia is associated with injury to sensory C-fiber neurons and little damage to A-fiber neurons.

The neuroimmune–glia interactions in the sensory ganglia have been firstly reported to account for the development of acute herpetic neuralgia by Silva et al. (34). Activation of glial cells and neuro-glial interactions are emerging as key mechanisms underlying the chronic pain. One type of glia cells, the astrocyte, is the main cell population type that composes the central nervous system. Increasing studies have shown that astrocytes are involved in the formation and development of the chronic neuropathic pain (68, 69), including PHN (28).

Activated by nitric oxide (NO), the increased expression of interleukin-1β in the astrocytes induced N-methyl-D-aspartic acid receptor (NMDAR) phosphorylation in spinal dorsal horn neurons to strengthen pain transmission (29). Although lncRNA is rarely reported in the PHN animal models, KCNA2-AS was found to be highly expressed in the spinal cord of PHN model rats. KCNA2-AS regulates PHN partly by combining with pSTAT3 to regulate cytoplasmic/nuclear translocation of pSTAT3, and then modulate NO-induced astrocyte activation, affecting the progression of PHN (25). This new finding provides a new insight on understanding the regulation mode of postherpetic pain.

### Ion channels

Abnormal expression of ion channels is also to blame for the neuronal hyperexcitability in PHN. The increased expression of α2δ1 calcium channel, Na(v)1.3 and Na(v)1.8 sodium channels has been proved in the DRG of the rats, which are thought to be related to VZV-induced neuropathic pain (29). In HSV1-induced pain models, TNF/TNFR1 signalling pathway mediates pain development by downregulating the inwardly rectifying K^+^ channel Kir4.1 in satellite glial cells and impairing K^+^ homeostasis (34).

### Pain-related genes

By gene array studies, the VZV-infected DRG showed changes in host gene expression patterns, with 84 up-regulated and 116 down-regulated genes, including nociception-associated genes *Ntrk2*, *Trpv1*, and *Calca* (CGRP) (24), which are validated to be associated with nociception.

## Therapeutic progress based on current rodent models

### Antiviral drugs only serve as acute stage of herpetic pain

Up to 90% of HZ patients experience acute pain, which may be alleviated by timely antiviral administration to limit viral replication (5). Providing antiviral agents, such as valaciclovir or famciclovir, early after onset of the rash has been shown to reduce VZV-induced pain. However, the therapeutic window is very short with treatment being effective when given within 72 h of the appearance of the rash (51), suggesting that the efficacy is due to a reduction in virus load. It is verified in the HSV 1-induced PHN mice model. On the vaccination day or the second day, intraperitoneal administration of 25 mg/kg acyclovir can inhibit hyperalgesia, rash and virus proliferation in the dorsal root ganglia of mice, but acyclovir has no effect on hyperalgesia from the fifth day.

### Classical analgesics only serve as acute stage of herpetic pain

Current analgesic treatment strategies for PHN include tricyclic antidepressants, topical lidocaine or capsaicin patch treatments, opioids and gabapentinoids (57).In the HSV 1 mice models, morphine (1-5mg/kg, subcutaneous injection), gabapentin (10-100mg/kg, perfusion), diclofenac (10-100 mg/kg, intraperitoneal injection) present a certain degree of analgesic effect (51, 64). Among them, morphine and gabapentin inhibited hyperalgesia in a dose-dependent manner. Mexiletine hydrochloride (30mg/kg, intragastric) and ketamine (50mg/kg, intraperitoneal injection) can also relieve HSV1-induced allodynia. Systemic administration of ibuprofen and diclofenac attenuate allodynia and hyperalgesia to a certain extent in VZV-inoculated rats. Given the key role of nitric oxide in inducing PHN, the allodynia can be inhibited by nitric oxide synthase inhibitors in the PHN rat models. Noteworthily, amitriptyline (10 mg/kg) markedly reduced the acute herpetic pain, but worked slower than gabapentin (47). This amitriptyline dosage produced a decreased tendency of PHN incidence, while gabapentin at the same dose failed. It is consistent with clinical report, but whether the suppression of late phase of acute pain correlated with the lower risk of PHN onset needs to be further explored (47).

While there is strong clinical evidence supporting the efficacy of certain analgesic therapies verified from rodent models, most of these therapies do suffer from a poor translation and narrow therapeutic index which limits their clinical effectiveness (10, 58).

### Other analgesic therapies

Proenkephalin is an endogenous opioid polypeptide hormone, which produces potentially analgesic molecules *via* certain biological processes (26). To produce a more long-term effect, the human proenkephalin gene (*vHPPE*) was expressed using a herpes simplex virus vector to deliver the gene in rats injected with VZV (26). The expression of *vHPPE* gene effectively reduces the pain sensitivity induced by VZV, and exerts analgesic effect for a long time in a dose-dependent manner (26). Hydrogen gas (H_2_) has been found to have anti-inflammatory and analgesic effects. Saline with a therapeutic dose of hydrogen (hydrogen-rich saline) is used to deliver molecular hydrogen (69). Intraperitoneal injection of 10 mL/kg of hydrogen-rich saline in PHN model rats for 7 days continuously reduced mechanical hyperalgesia and inflammatory factors autophagy by activating autophagy (70). However, the administration route of hydrogen-rich saline in patients and how to ensure the functional hydrogen molecules reach the affected tissues (spinal cord or dorsal root ganglia) of PHN patients remain elusive. There is still a long way to go to apply these therapies in PHN patients.

## Conclusion

In the past two decades, rodent models mimicking PHN have been established and the nociception in the rodents have been evaluated by the observing their certain behaviour (**Table 1**). In sum, no single model can capture the full spectrum of clinical features of PHN, and no single assay can fulfil the comprehensive assessment in the non-communicating animals. Thus, we need to establish an overview combining different PHN models to design the experiments and select appropriate models carefully and rationally. For the two rodent models discussed above, the significant difference is whether the nervous system allows virus proliferation. Overall, the HSV1-induced pain model can be used not only for studying the pathogenesis of PHN and screening related antiviral drugs as well as analgesics, but also for other viruses with neurotropic properties, such as polio virus, rabies virus, etc. Although the VZV-induced rat models lack some clinical manifestation like skin rash and virus replication, the allodynia and hyperalgesia caused by the latent infection of VZV persisted, offering a potential for studying the mechanism of occurrence and development of postherpetic neuralgia and finding new analgesic targets.

Looking back the development of PHN models, one of the main difficulties is the strict human specificity of VZV. Similar neurotropic viruses, such as HSV1, have been attempted to establish models for PHN study. However, different viruses may influence herpetic pain *via* different biological pathways. Due to VZV is the only pathogen causing PHN, how to overcome the specificity by using VZV strains in rodent models is far from full consideration. The cellular receptor insulin-degrading enzyme (IDE) has been identified to be essential for VZV infection and spread through recognizing VZV glycoprotein E (71). Apart from SCID-hu model, receptor humanization in rodent animals also have the potential to overcome the species specificity of VZV.

Besides, other than reactivation, the pain of these two models is based on acute VZV infection, deviating from the normal route of PHN progression in humans. To date, no animal model of VZV reactivation has been developed. The combination of body irradiation and immune suppressant regimens have been attempted to induce virus reactivation in SVV-infected models (15), which provide a direction to further optimize VZV or HSV1 induced herpetic pain model. If an effective model of virus reactivation could be developed and displayed PHN-like herpetic pain, it will fill in the gap of unknown relationship between PHN and virus latency/reactivation.

In addition, a new model has been employed to mimic the unique sensation changes of PHN patients by single intraperitoneal injection of RTX (200 or 250 μg/kg) to avoid virus infection, which requires rigorous experiment environment, such as isolated rooms for virus growth and injection (52). Some PHN patients displayed increasing mechanical hyperalgesia but lower thermal hypoalgesia (less sensitive to heat) (72). This unique behavioural sign has been reported to be observed in RTX models, thus provide a useful tool for understanding the mechanism behind the unique clinical features and screening out targeted drugs.

As for the pain evaluation in PHN rodent models, it can be more comprehensive in the future, focusing more on the non-stimuli responses after inducing neurological pain. Besides, functional imaging in unanaesthetised PHN models is extremely rare, as most of pain researches in humans derives from imaging studies (73). Developing and attempting new evaluation methods closer to human pain may help us improve our knowledge on PHN and screening targeted therapies.

## Author contributions

MO: Drafting/revision of the manuscript for content, including writing for content; Analysis or interpretation of data. JC: Drafting/revision of the manuscript for content, including writing for content; Major role in the acquisition of data. SY: Drafting/revision of the manuscript for content, including writing for content; Analysis or interpretation of data. LX: Study concept or design; Analysis or interpretation of data. DX: Study concept or design. SW: Drafting/revision of the manuscript for content, including writing for content; Study concept or design; Analysis or interpretation of data. All authors contributed to the article and approved the submitted version.
